# A Statistical Estimation Approach for Quantitative Concentrations of Compounds Lacking Authentic Standards/Surrogates Based on Linear Correlations between Directly Measured Detector Responses and Carbon Number of Different Functional Groups

**DOI:** 10.1155/2013/241585

**Published:** 2013-05-12

**Authors:** Yong-Hyun Kim, Ki-Hyun Kim

**Affiliations:** Atmospheric Environment Laboratory, Department of Environment & Energy, Sejong University, Seoul 143-747, Republic of Korea

## Abstract

A statistical approach was investigated to estimate the concentration of compounds lacking authentic standards/surrogates (CLASS). As a means to assess the reliability of this approach, the response factor (RF) of CLASS is derived by predictive equations based on a linear regression (LR) analysis between the actual RF (by external calibration) of 18 reference volatile organic compounds (VOCs) consisting of six original functional groups and their physicochemical parameters ((1) carbon number (CN), (2) molecular weight (MW), and (3) boiling point (BP)). If the experimental bias is estimated in terms of percent difference (PD) between the actual and projected RF, the least bias for 18 VOCs is found from CN (17.9 ± 19.0%). In contrast, the PD values against MW and BP are 40.6% and 81.5%, respectively. Predictive equations were hence derived via an LR analysis between the actual RF and CN for 29 groups: (1) one group consisting of all 18 reference VOCs, (2) three out of six original functional groups, and (3) 25 groups formed randomly from the six functional groups. The applicability of this method was tested by fitting these 29 equations into each of the six original functional groups. According to this approach, the mean PD for 18 compounds dropped as low as 5.60 ± 5.63%. This approach can thus be used as a practical tool to assess the quantitative data for CLASS.

## 1. Introduction

Gas chromatographs (GCs) equipped with either a flame ionization detector (FID) or mass spectrometer (MS) as a detector have been most commonly used for the analysis of diverse organic compounds in environmental media [1, 2]. If the MS uses 70 eV EI ionization, the RF (per unit weight) should be generally constant within each compound class (e.g., linear increase of the response with MW) [3]. The quantitative analysis of the reference compounds can be made in the form of the external calibration using standards containing each reference compound. If the number of target compounds contained in standard is limited, it is difficult to derive quantitative data for some important compounds without proper standards material (i.e., standards for many chemicals cannot be available commercially) or to the complexity involved in standard preparation. The number of possible compounds and their isomers rapidly increases with increasing MW, and consequently, synthesis of all proper standards is unrealistic except possibly for the lowest MW analytes. Thus, the development of predictive algorithms for the compounds lacking authentic standards/surrogates (CLASS) is one realistic option to assign semiquantitative values for such chemical species.

Ahn et al. [4] investigated a method for predicting the concentration of various CLASS using liquid phase standards of 54 VOCs based on two contrasting approaches: (1) direct injection (DI) and (2) headspace solid-phase microextraction (HS-SPME). Because the DI approach does not involve any necessary pretreatment procedures for environmental samples, its practicality can be significantly restricted. On the other hand, as the HS-SPME approach is subject to relatively low recovery, it is difficult to quantify trace level samples with reasonable reliability [5]. In addition, as 54 reference VOCs of Ahn et al. [4] basically represent the water quality index, the applicability of such an approach requires testing against chemicals with different characteristics.

In order to extend our efforts to estimate CLASS quantitatively, we explored the reliability of our early model introduced by Ahn et al. [4] by fitting it against several representative odorous VOC classes with the modification of the grouping approaches. Moreover, to widen the applicability of this statistical approach to the trace-level analysis of environmental samples, we conducted a series of validation tests to assure the experimental reliability of our approach based on the calibration experiments with combination of the sorbent tube (ST) and thermal desorption (TD) methods [6, 7]. Through a comparative analysis between the measured and predicted RF values of the selected reference compounds, the reliability of this modified estimation approach has been examined in a number of respects. After all, we present a rough but systematic solution for quantification of CLASS in environmental media based on the predictive equations developed in this preliminary study.

## 2. Materials and Methods

### 2.1. Experimental Approaches

To generate predictive equations for the projected RF value of CLASS, a linear plot was drawn by considering the relationship between the directly measured (actual) RF values of reference odorous VOCs (mostly with low odor threshold) and their physicochemical parameters (e.g., carbon number (CN)). Then, the projected RF values for each compound were evaluated for reliability by direct comparison against the actual RF values. 

Table 1 shows the conceptual schematic of the experimental approaches used in this study in reference to the previous study of Ahn el al. [4]. As the experimental approach used in this study has been considerably modified from that of the study of Ahn et al. [4], we reevaluated the feasibility of that study as the major reference during the course of this study. To facilitate this reevaluation, the two data sets obtained by Ahn et al. [4] ((1) direct injection of liquid standard (DILS) and (2) solid-phase microextraction (SPME)) were referred to as “Exp-DI” and “Exp-SPME,” respectively. In contrast, as the analytical method used in the present study was based on the thermal desorption (TD) method, it was named “Exp-TD” for comparison with the two previous approaches. 

### 2.2. Selection and Preparation of Working Standards

A total of 19 VOCs were initially selected as the reference analytes for this study: (1) five aldehydes: acetaldehyde (AA), propionaldehyde (PA), butyraldehyde (BA), isovaleraldehyde (IA), and n-valeraldehyde (VA); (2) six aromatics: benzene (B), toluene (T), styrene (S), *p*-xylene (p-X), *m*-xylene (m-X), and *o*-xylene (o-X); (3) four carboxylic: propionic acid (PPA), butyric acid (BTA), isovaleric acid (IVA), and n-valeric acid (VLA); (4) two ketones: methyl ethyl ketone (MEK), and methyl isobutyl ketone (MIBK); (5) one alcohol: isobutyl alcohol (i-BuAl); and (6) one ester: n-butyl acetate (BuAc) (Table 2). For the reader's reference, all these selected VOCs have low odor thresholds except for benzene [8, 9]. Primary-grade chemicals containing these VOCs were purchased at the purity of ≥97%. The liquid-phase working standards (L-WS) were prepared by a gravimetric dilution of the primary-grade chemicals using methanol. Table 1S shows the detailed procedures for making the L-WS (see Supplementary Material available on line at http://dx.doi.org/10.1155/2013/241585). The basic information of 54 reference VOCs selected by Ahn et al. [4] for Exp-DI and -SPME is also provided in Table 2S. The procedures used for the preparation of those working standards have been described elsewhere [4]. The 19 VOCs selected in this study have several functional groups. However, as the number of these target compounds is limited, we have a plan to add more reference compounds to expand the applicability of our method as well as its validation.

### 2.3. Instrumental System

The analysis of VOC samples in this study was carried out using GC (Shimadzu GC-2100, Japan) equipped with MS (Shimadzu GCMS-QP2010, Japan) and a UNITY thermal desorber (Markes International, Ltd, UK). The sorbent tube, filled with each 100 mg of Tenax TA, Carbopack B, and Carboxen 1000, was used as the collection media to preconcentrate the L-WS. The cryofocusing trap in the TD unit was packed withTenax TA and Carbopack B at a 1 : 1 volume ratio (inner diameter = 2 mm and total sorbent bed length = 50 mm). The reference VOCs were separated on a CP-Wax column (diameter = 0.25 mm, length = 60 m, and film thickness = 0.25 *μ*m) with a 50 min running cycle. Detailed information of instrumental system is described in Table 3.

### 2.4. Data Acquisition and Quality Assurance

In this study, the calibration data for all reference VOCs were drawn by their L-WS with the aid of the sorbent-based tube approach. For quantification of CLASS, we have to use the TIC mode. This is because the RF patterns of CLASS against their CN of VOC can be only obtained by the full scan mode (TIC mode) in MS system. If we select the SIM mode (selected the spectrum), we cannot find the RF patterns of CLASS. Although it is not easy to separate diverse compounds using GC system, we can technically separate not all but many diverse compounds by controlling the TD-GC conditions. Details of the ST approach have been described elsewhere [10]. The L-WS was injected directly into the sampling inlet of the ST using a microsyringe, while a flow of inert back-up gas was constantly maintained (flow rate = 50 mL min^−1^ for 10 min). Once the ST was loaded by the L-WS containing 19 reference VOCs, it was then analyzed by the TD-GC-MS system. Two calibration experiments were conducted using the L-WS prepared at five concentration levels (in case of benzene: 1.22, 6.12, 12.2, 24.5, and 61.2 ng *μ*L^−1^). The reproducibility of experimental data was assessed in terms of relative standard errors (RSE; %) using triplicate analyses of the forth calibration point (24.5 ng *μ*L^−1^ in case of benzene). Detectability of reference compounds was calculated as the method detection limits (MDLs) by following the relevant US EPA guidelines. Seven repeated analyses were made using the standard of the 61.2 pg *μ*L^−1^ (in case of benzene), which was obtained by diluting the standard of the first calibration point (Table 1S). The resulting SD values were then multiplied by 3.14 (Student's *t*-value at the 99.9% confidence interval) to yield the MDL in mass quantity (pg), which ranged from 10.1 pg (o-X) to 3,878 pg (AA) (mean 235 ± 883 pg).

## 3. Result and Discussion

### 3.1. Development of Predictive Equations for Class by Linear Regression (LR) Analysis

In this study, calibration of liquid-phase standards containing 19 reference compounds was initially conducted. The basic calibration data (RF, *R* ^2^, and RSE (%)) for each individual compound were obtained from replicate calibration experiments of the L-WS containing those VOCs. As shown in Table 4, all coefficients of variation (CV, %) for RF were fairly constant and low for all VOCs with their mean CV (*n* = 19) = 1.30 ± 1.12%. As such, the calibration results of all VOCs were fairly stable and reproducible. 

As a simple means to develop predictive equations for CLASS, a linear regression (LR) analysis was made between the actual RF and the three key parameters consisting of (1) CN, (2) MW, and (3) BP. As a means to obtain the best predictive equations, we attempted to establish the key variables for the equations. To this end, the compatibility of each of all three key variables was initially checked between their directly measured (actual) and projected RF values (Table 5). In addition, another type of test was also conducted in the next section in which such compatibility is also evaluated through grouping of all target compounds into various segments to yield the best match between prediction and real measurements (Table 6). In our laboratory, we are currently developing the methods to test the reliability of CLASS method by measuring standards containing chemicals that do not match with target compounds used to derive predictive equations [11]. In the meantime, the validity of our methodology in this work can be optimized through a number of indirect evaluation procedures described in Tables 5 and 6. Because of the unusually low recovery pattern, the predicting equations were however developed using 18 instead of all 19 reference compounds after excluding AA. The unfeasibility of the ST/TD approach for AA had already been demonstrated in our recent study [10]: the RF value of AA is considerably lower (9 to 55 times) than other aldehydes (AA = 1,854, PA = 16,117, BA = 78,028, IA = 102,217, and VA = 88,556). Hence, all of our predictive equations are tested based on 18 reference compounds (without AA).

Table 5 shows the predictive equations derived by an LR analysis of 18 reference VOCs between the actual RF versus three key parameters along with their percent difference (PD) values between the actual and projected RF:

(1) PD  value (%) =|(RF (Projected)−RF (Actual))|RF (Actual)∗100.

In Table 5, the projected RF values for each of all 18 reference compounds are summarized for a whole group and three functional groups, the aldehyde, aromatic, and carboxylic groups. If the PD values were calculated using the equations for all the reference VOCs (*n* = 18) as a single group, the least magnitude of PD (or least bias) was found from the CN (17.9 ± 19.0%). In contrast, the results for MW and BP were 40.6 ± 27.4% and 81.5 ± 112%, respectively. In addition, a comparison of the coefficient of determination (*R* ^2^) showed that the CN had the highest *R* ^2^ value of 0.9396, while the MW and BP were 0.5445 and 0.1404, respectively. It is difficult to derive the predictive equation for quantification of CLASS using only the physicochemical parameters because of such biases in their predictive equations. 

**Figure 1:**
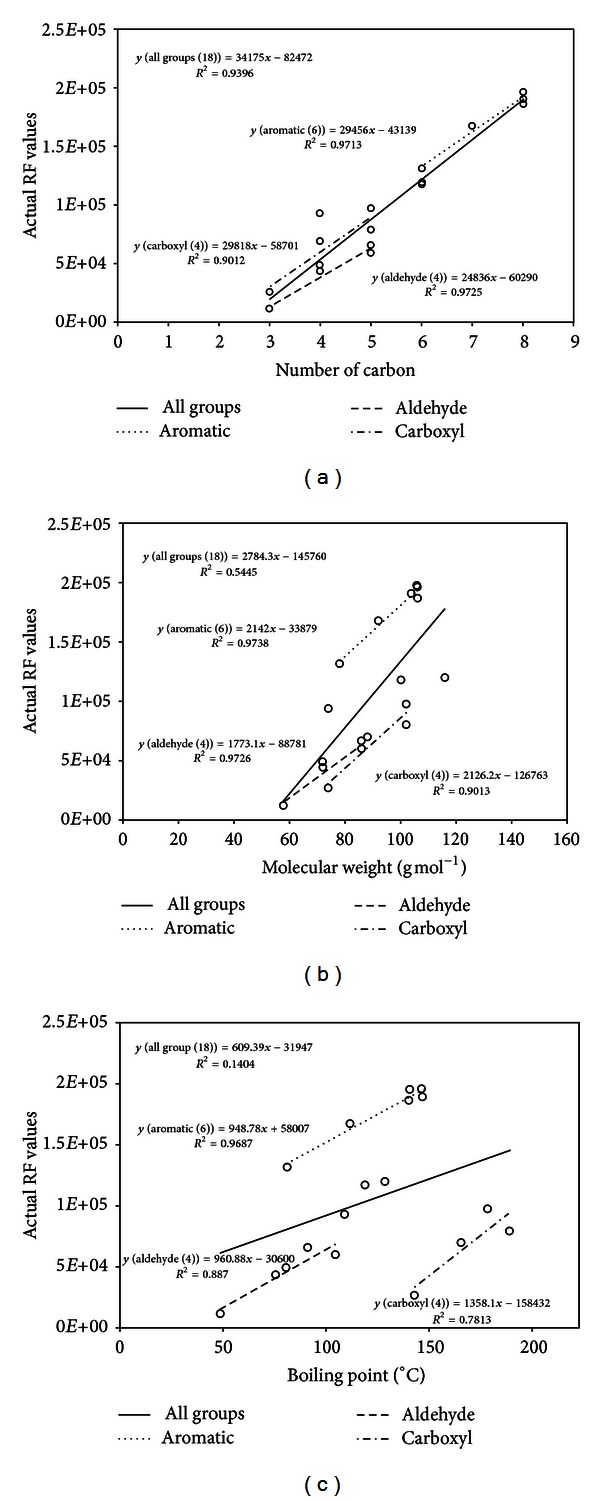
The correlations between RF values and key variables: (a) carbon number, (b) molecular weight, and (c) boiling point in Exp-TD.

To find the possibly least biased predictive equations, the PD values and *R* ^2^ values derived from 18 VOCs were examined as a whole and three functional groups (aldehyde (*n* = 4), aromatic (*n* = 6), and carboxylic (*n* = 4)). The results shown in Table 5 indicate that the PD and *R* ^2^ values of the three functional groups can be improved further than those derived from 18 VOCs as a whole. For instance, the results of three groups against CN showed PD values below 9.83 ± 6.55%, 2.03 ± 0.76%, and 12.7 ± 4.30%, respectively (with all their *R* ^2^ values above 0.9). In addition, the PD values examined in terms of MW and BP also improved significantly. In the case of the former, the PD values varied between 1.73% and 12.7%, while those of the latter were between 2.08% and 17.7% (Table 5). Their *R* ^2^ values also ranged from 0.9013 to 0.9738 and 0.7813 to 0.9687, respectively. It means that the RF values of VOCs are significantly associated with their physicochemical parameters like MW and BP if the VOCs belong to same functional groups. The projected equations for each functional group are also plotted in Figure 1. 

### 3.2. LR Analysis against CN with Arbitrary Chemical Grouping

As mentioned in Section 3.1, the projected RF values of the reference VOCs were first derived by an LR analysis between actual RF and their three key parameters. As a result, we found that the CN (among all three physical factors) yielded the optimal (minimum) PD values (between actual and projected RF values). However, the results for MW and BP also improved, if the analysis is made after being divided into a number of small groups. Hence, to develop the predictive equations with the least bias (or the smallest PD values), classification of our 18 reference compounds was made further to yield a total of 29 VOC groups, as shown in Table 6. These 29 groups consist of (1) one group consisting of all 18 compounds, (2) three (out of the original six) functional groups consisting of more than 4 compounds, and (3) 25 arbitrary groups formed randomly by the combination of the original 6 functional groups.

The sorting scheme for the aforementioned third case (*n* = 25) can be explained as follows. If three functional groups (aldehyde, aromatic, and carboxylic) each of which consist of more than four compounds are referred to as “Major,” then the rest of the original functional groups (ketone, alcohol, and ester) with less than 2 compounds are referred to as “Minor.” Hence, the combination of these major and minor groups was made to produce the following 25 different arbitrary groups:

- Major (single pair) (I or II or III) + Major (single pair) (IV or V or VI) = 9,
- Major (double pair) (I + II or II + III or I + III) + Minor (single pair) (none or either of IV, V, or VI) = 12,
- Major (triple pair) (I + II + III) + Minor (single pair) (none or either of IV, V, or VI) = 4.

Note that the CN was used as the main variable to obtain the predictive equations. Thus, we computed the projected equations for each of these 29 VOC groups as a function of the CN (Table 6). 

If each individual of all 29 predicting equations are tested against the total 18 reference compounds as a whole (like a single group), the 29 resulting PD values averaged 14.9 ± 9.90% (Table 6). The mean *R* ^2^ of all projected equations yielded a fairly high value of 0.8955 ± 1.490 (*n* = 29). Likewise, the 29 predicted equations can also be fitted against each of the six main VOC functional groups of I through VI in Table 6. As we eventually intend to test the applicability of this predictive equation to any CLASS by the identity of its functional group, we attempted to find the best fitting equation by testing all 29 equations to each of the six original functional groups that were sorted from 18 reference compounds. According to this approach, the patterns of PD computation contrasted in two different ways. At first, the projected RF values of aromatic (II), ketone (IV), and ester (VI) were almost similar to their actual RF values derived by the L-WS, yielding mean PD values below 10% (aromatic (*n* = 17) = 3.26 ± 0.74%, ketone (*n* = 8) = 8.95 ± 5.36%, and ester (*n* = 8) = 4.61 ± 3.67%). In contrast, aldehyde (I) and alcohol (V) had relatively high PD values of 33.0 ± 12.1% (*n* = 17) and 34.1 ± 14.0% (*n* = 8), respectively. Hence, if the minimum PD values derived by the 29 equations are taken for each of the six groups, the minimum PD values of the six functional groups were greatly reduced (e.g., below 10% in range of 0.27% (ester) to 9.87% (carboxylic) (Table 6).

Table 7 shows the results of a stepwise approach to find the optimal PD of the 18 reference compounds: (1) by matching the best equation for a given functional group and (2) by computing the PD value for each member of each functional group by the equation selected in the first step. As we intend to find and apply the predictive equations for CLASS, we selected the best equations for each of all six functional groups that comprise 18 reference compounds. As shown in Table 7, if the best fit equations for each of the six classes are applied, the PD values for all 18 compounds are dramatically reduced (5.60 ± 5.63%). The feasibility of this grouping scheme on the estimation bias can be tested as follows. If classification of the 18 reference compounds is made in a simple form, for example, three groups instead of six groups, the PD values were again raised near 10% from 5% level (Table 3S). Thus, by following this type of procedure to match the best equation for each member of the original functional groups, one is able to find the optimal projected equation for any CLASS, once it is identified as the member of a certain functional group. 

### 3.3. Comparison of the Projected RF Values with the Previous Study

In this section, we investigated a simple basic means to test the validity of four predictive equations using the data sets made from other studies (e.g., [4, 11]). Through the re-evaluation of those previous studies, we explored the validity of our new approach—whether the RF patterns of VOCs against their CNs (for a given VOC group) were formed or not. In the previous study of Ahn et al. [4], some predictive methods were investigated to estimate the concentrations of various CLASS using liquid-phase standards containing 54 VOCs based on the two contrasting experiment approaches of Exp-DI and Exp-SPME (Table 4S). However, the calibration data of Ahn et al. [4] were evaluated in a simplified way without considering chemical grouping of CLASS. Unlike the case of this study. The results of Ahn et al. [4] were hence reinterpreted to allow comparison with this study on a parallel basis as follows.

The raw calibration data of the Exp-DI and -SPME of Ahn et al. [4] presented in Table 3 yielded mean *R* ^2^ values of 0.9666 ± 0.0275 (*n* = 51) and 0.9856 ± 0.0410 (*n* = 50), respectively. The two methods investigated previously also had a good analytical reproducibility (RSE for all below 3%). Out of 54 compounds analyzed in the previous study, we focused on 49 compounds, excluding five (1,2-dichloroethane, 1,1,1,2-tetrachloroethane, methylene chloride, *p*-xylene, and hexachlorobutadiene) for a parallel comparison (due to the detectability or eccentricity of calibration problems). 

To make a meaningful evaluation of the Ahn et al. data, LR analysis was also conducted against three parameters ((1) CN, (2) MW, and (3) BP). In Table 5S, the projected RF values of 49 reference VOCs consistently had the least mean PD values with CN for both experimental methods: (1) Exp-DI (*n* = 49): PD (CN) = 31.3 ± 38.7%, PD (MW) = 89.5 ± 83.0%, and PD (BP) = 52.8 ± 70.9% and (2) Exp-SPME (*n* = 49): PD (CN) = 44.0 ± 95.3%, PD (MW) = 182 ± 263%, and PD (BP) = 127 ± 196%) (Figure 1S).

If the projected equations were derived after dividing all chemicals into multiple VOC functional groups, the PD values against three physical parameters improved further into a narrow range. For instance, in the case of Exp-DI, the mean PD results of haloalkane (*n* = 17) fell in the range of 30.2% (BP) to 35.9% (MW), while those of aromatic (*n* = 24) fell in the range of 28.3% (CN) to 31.2% (MW). In the case of Exp-SPME, the mean PD results of haloalkane (*n* = 17) fell in the range of 48.7% (BP) to 50.5% (MW), while those of aromatic (*n* = 24) changed from 11.8% (CN) to 20.3% (MW). 

To derive the best predictive equations using the data sets of Ahn et al. [4], we checked the compatibility between the two RF values from the maximum numbered (13) groups of 49 reference VOCs: (1) one group consisting of all 49 compounds, (2) two functional groups (multiple components with different CNs), and (3) 10 arbitrary groups randomly formed by the combination of the four original VOC functional groups (Table 6S). Then, the PD values were computed for the whole component (as a single group) and each of all four VOC groups by fitting the 13 predictive equations. However, in comparison with the present study, the Exp-DI and -SPME generally had weak linearity with mean *R* ^2^ values of 0.4576 and 0.4565, respectively; these values were far lower than the Exp-TD counterpart (0.8955) in this work. The minimum PD values of all reference VOCs of Ahn et al. [4], if assessed by the best fit equations, averaged between 27.5 ± 34.2% (Exp-DI) and 30.3 ± 45.3% (Exp-SPME) (Table 7S). Although the number of reinvestigated model compounds (*n* = 49) is much larger than that of this study (*n* = 18), the compatibility of RF values in the previous study is dramatically low relative to this study (Exp-TD = 5.60 ± 5.63%).

In line with our efforts to develop estimation methods, Allgood et al. [12] also attempted to predict the concentration of CLASS in complex mixtures. These authors examined the ratio of relative sensitivity (comparable to RF in this work) among the 43 reference compounds (VOCs) against their MWs. The RF values of all reference compounds measured by GC-MS system yielded two types of calibration results: (1) molar sensitivity and (2) mass sensitivity. More specifically, these authors used the mole and mass values of each reference compound as the variable of the *x*-axis to derive sensitivity (or RF) (*y*-axis = peak area). The relative molar and mass sensitivities of all reference compounds were then calculated against n-octane as the key reference (relative sensitivity = reference compounds sensitivity/n-octane sensitivity). 

**Figure 2:**
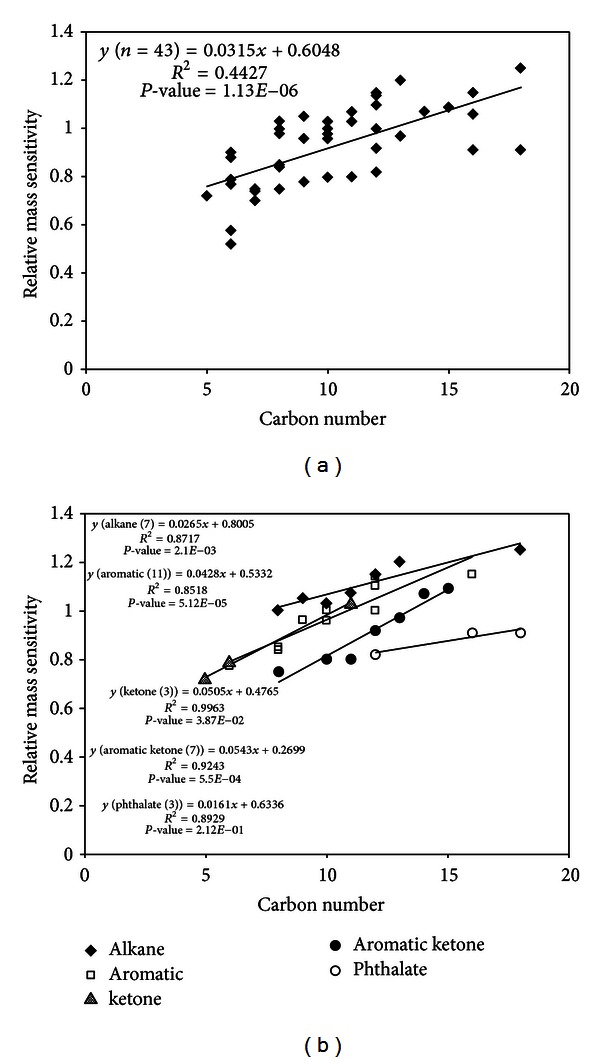
Reinterpretation of the mass sensitivity data by Allgood et al. [12] against carbon number.

Allgood et al. [12] found that the relative molar sensitivities yielded a stronger linear correlation with the MW (*R* ^2^ = 0.7878) than with the relative mass sensitivity (*R* ^2^ = 0.0968). Although these authors assessed sensitivity only in relation to molar and mass parameters, their results can also be reevaluated by adopting our approach, wherein the CN is used as the key criteria instead of MW. Hence, the data of Allgood et al. [12] are newly examined by the same procedure of this study. To derive the best results based on our approach, all chemicals (*n* = 43) tested by Allgood et al. [12] were grouped into five functional groups and plotted against CN. As expected, the *R* ^2^ values then improved significantly (*R* ^2^ and *P* values = 0.8717 and 2.10*E* − 03 (haloalkane, *n* = 7), 0.8518 and 5.12*E* − 05 (aromatic, *n* = 11), 0.9963 and 3.87*E* − 02 (ketone, *n* = 3), 0.9243 and 5.50*E* − 04 (aromatic ketone, *n* = 7), and 0.8929 and 2.12*E* − 01 (phthalate, *n* = 3)). As such, these obtained results are far better than the original results simply examined against MW (*R* ^2^ = 0.4427 and *P* value = 1.13*E* − 06) (Figure 2). The results of our comparative efforts to reevaluate the two previous studies [4, 12] consistently confirm that the use of the modified statistical approach tested in this study can be used to produce improved predictions of CLASS in relation to selected reference chemical group.

## 4. Conclusions

The external calibration has been the most commonly used method for the quantitative analysis of diverse VOCs in environmental media. If the number of detected VOCs exceeds hundreds to thousands, assessment of all individual components in a quantitative sense is not easy. This is because all detected VOCs cannot be standardized (because of unavailability of a standard material, etc.). In this study, 18 VOCs representing the six functional groups were selected as the reference to develop predictive equations to assess the concentrations of CLASS belonging to any of those functional groups. To find the optimal predictive equations of each reference compound, we conducted a series of LR analyses between their actual RF values (derived by external calibration of the liquid standard by the sorbent tube method) and three physicochemical properties (of the model compounds): (1) CN, (2) MW, and (3) BP.

As a means to validate the applicability of the predicting equations for CLASS, a total of 18 reference VOCs were arbitrarily classified into 29 VOC groups by the combination of the raw six functional groups. Then, the reliability of this approach was evaluated by assigning the six best fit equations to each of all six groups and examined in terms of the PD value between different RFs. If the optimal PD values of each reference compound are derived for each of all 18 compounds, they averaged as low as 5.60 ± 5.63% (range of 0.27% (Ester) to 18.6% (PA)). As a result, we were able to demonstrate the possibility that the projected RF values of the 18 reference VOCs, if assessed by this statistical approach, can comply well with their actual RF values determined experimentally. In other words, if the predictive equations were used to estimate the concentration of CLASS in real environmental samples, it is possible to derive quantitative concentration data for the CLASS with a fairly low experimental uncertainty.

In conclusion, if one can obtain and carry out the external calibration for a number of VOCs with diverse chemical functional groups, those calibration data may be used to develop a predictive equation to quantitatively determine CLASS with reasonably good confidence. As a new task, we are currently putting extensive efforts to validate the experimental performance of this approximation tool against various standard mixtures or real environmental samples. The results of this validation approach will soon be reported. In the meantime, the results obtained in this preliminary study will be able to offer valuable insights into the potential use of this predictive approach for various CLASS.

## Supplementary Material

Table 1S: Preparation of liquid phase standard of 19 VOC for the analysis by the TD-GC-MS based analysis in this study.
Table 2S: List of the 54 VOCs investigated in previous study (Exp-DI and -SPME) by Ahn et al. [4].
Table 3S: Results of the projected RF values derived by simplification of projected grouping.
Table 4S: Operational conditions of GC-MS system for the analysis of reference VOCs in previous study of Ahn et al. [4].
Table 5S: Comparison of percent difference (PD) between actual and predicted response factor (RF) values for all (n=49) or two chemical groups (n=41) in relation to three major variables of reference VOCs used for prediction: (1) carbon number, (2) molecular weight, and (3) boiling point (Ahn et al. [4]).
Table 6S: Assessment of the least PD values between the actual and projected RF values for arbitrarily divided chemical groups based on linear regression analysis using Exp-DI and-SPME data (Ahn et al. [4]).
Table 7S: Results of the minimum percent difference (PD) values between the best projected and actual RF in the study of Ahn et al. [4].
Fig. 1S: The correlations between RF values and key variables (A. carbon number, B. molecular weight, and C. boiling point) in Ahn et al. [4].

## Figures and Tables

**Table 1:** Conceptual schematic of experimental (Exp) approaches to estimate response factor (RF) values of compounds lacking authentic standards/surrogates (CLASS).

Order	Type^a^	This study	Previous study (reference)	
		Exp-TD	Exp-DI	Exp-SPME
1	Reference	This study	Ahn et al. (2011) [4]	Ahn et al. (2011) [4]

2	Standard	Lab-made mix	The 502/524 volatile organics calibration mix	The 502/524 volatile organics calibration mix
		(Primary grade chemical: Sigma-Aldrich, USA)	(Supelco, St. Louis, MO)	(Supelco, St. Louis, MO)

3	Compounds	18 compounds are used among 19 compounds (a list of offensive odorants plus a few reference compound)	49 compounds are analyzed among 54 compounds (haloalkane, chloropropene, chloroethene, aromatic, and diene)	49 compounds are analyzed among 54 compounds (haloalkane, chloropropene, chloroethene, aromatic, and diene)

4	Method of calibration	Injection of liquid standard on sorbent tube and thermal desorption analysis (ST analysis)	Direct injection of liquid standard (DILS)	Vapor phase injection via SPME (SPME)

5	Calibration results^b^	*R* ^2^ = 0.9954 ± 0.0075 (*n* = 19)	*R* ^2^ = 0.9963 ± 0.0278 (*n* = 51)	*R* ^2^ = 0.9854 ± 0.0414 (*n* = 50)
		RSE = 1.39 ± 0.82 (*n* = 19)	RSE = 2.85 ± 2.26% (*n* = 51)	RSE = 1.59 ± 1.17% (*n* = 49)

6	Percent difference (PD) between actual versus best projected RF	5.60 ± 5.63% (*n* = 18)	27.5 ± 34.2% (*n* = 49)	30.3 ± 45.3% (*n* = 49)

^a^Pretreatment approaches: (1) Exp-TD: thermal desorption, (2) Exp-DI: direct injection, and (3) Exp-SPME: solid-phase microextraction (SPME).

^b^Comparison of mean values of correlation coefficients and relative standard error (RSE) derived for each individual compound using each calibration method.

*Same experimental conditions between this and a previous study.

(1) Raw standard phase: liquid phase.

(2) Method of detection: GC (Shimadzu GC-2010, Japan) and MS (Shimadzu GCMS-QP2010, Japan).

**Table 2:** List of 19 VOCs investigated in this study for the derivation of predictive equations for CLASS.

Order	Group	Compounds^a^	Short name	Carbon numbers	MW (g mol^−1^)	Density (g cm^−3^)	Boiling point (°C)	Formula	CAS number
1	Aldehyde	Acetaldehyde	AA	2	44.1	0.785	20.2	C_2_H_4_O	75-07-0
2		Propionaldehyde	PA	3	58.1	0.798	46–50	C_3_H_6_O	123-38-6
3		Butyraldehyde	BA	4	72.1	0.805	74.8	C_4_H_8_O	123-72-8
4		Isovaleraldehyde	IA	5	86.1	0.797	90	C_5_H_10_O	590-86-3
5		n-Valeraldehyde	VA	5	86.1	0.81	103	C_5_H_10_O	110-62-3

6	Aromatic	Benzene	B	6	78.11	0.878	80.1	C_6_H_6_	71-43-2
7		Toluene	T	7	92.14	0.866	110.6	C_7_H_8_	108-88-3
8		Styrene	S	8	104.2	0.906	145	C_8_H_8_	100-42-5
9		p-Xylene	p-X	8	106.2	0.865	138.35	C_8_H_10_	106-42-3
10		m-Xylene	m-X	8	106.2	0.865	139	C_8_H_10_	108-38-3
11		o-Xylene	o-X	8	106.2	0.88	144.4	C_8_H_10_	95-47-6

12		Propionic acid	PPA	3	74.1	0.99	141	C_3_H_6_O_2_	79-09-4
13	Carboxylic	n-Butyric acid	BTA	4	88.1	0.958	163.5	C_4_H_8_O_2_	107-92-6
14	(volatile fatty acid)	i-Valeric acid	IVA	5	102	0.925	175–177	C_5_H_10_O_2_	503-74-2
15		n-Valeric acid	VLA	5	102	0.938	186-187	C_5_H_10_O_2_	109-52-4

16	Ketone	Methyl ethyl ketone	MEK	4	72.11	0.805	79.64	C_4_H_8_O	78-93-3
17		Methyl isobutyl ketone	MIBK	6	100.2	0.802	117-118	C_6_H_12_O	108-10-1

18	Alcohol	Isobutyl alcohol	i-BuAl	4	74.12	0.801	107.89	C_4_H_10_O	78-83-1

19	Ester	n-Butyl acetate	BuAc	6	116.2	0.881	127	C_6_H_12_O_2_	123-86-4

^a^18 compounds except for AA are used to calculate the projected response factor (RF) for model compounds.

**Table 3:** Operational conditions of the TD-GC-MS system for the analysis of reference VOCs in this study.

(A) GC (Shimadzu GC-2010, Japan) and MS (Shimadzu GCMS-QP2010, Japan)			
Column: CP Wax (diameter: 0.25 mm, length: 60 m, and film thickness: 0.25 *μ*m)			
Oven setting		Detector setting	

Oven temp.	35°C (10 min)	Ionization mode	EI (70 eV)
Oven rate	6°C min^−1^	Ion source temp.	200°C
Max oven temp.	215°C (10 min)	Interface temp.	200°C
Total time	50 min	TIC scan range	35~260 m z^−1^
Carrier gas	He (99.999%)	Carrier gas flow	1 mL min^−1^

(B) Thermal desorber (Unity, Markes, UK)			

Cold trap sorbent	Carbopack C + Carbopack B (volume ratio = 1 : 1)		
Split ratio	1 : 5	Adsorption temp.	−10°C
Split flow	5 mL min^−1^	Desorption temp.	320°C
Trap hold time	20 min	Flow path temp.	150°C

(C) Sorbent (sampling) Tube			

Sorbent material	Tenax TA + Carbopack B + Carboxen 1000 (mass (mg) = 100 : 100 : 100 )		
Desorption flow	50 mL min^−1^		
Desorption time	5 min	Desorption temp.	300°C

**Table 4:** Results of replicate calibration of 19 reference VOCs based on ST/TD approach used in this study: (1) response factor (RF), (2) determination of coefficient (*R* ^2^), and (3) relative standard error (RSE, %).

Order	VOC group	Compound	Actual RF				*R* ^2^			RSE^b^ (%)
			1st	2nd	Mean	CV^a^	1st	2nd	Mean	
1	Aldehyde	AA	522	497	**510**	3.47	0.9619	0.9698	0.9659	2.52
2		PA	12,017	11,950	**11,984**	0.40	0.9991	0.9991	0.9991	3.49
3		BA	43,572	43,467	**43,520**	0.17	0.9963	0.9938	0.9951	1.05
4		IA	66,125	65,836	**65,981**	0.31	0.9962	0.9932	0.9947	1.93
5		VA	59,322	59,804	**59,563**	0.57	0.9973	0.9973	0.9973	1.35

6	Aromatic	B	131,760	131,280	**131,520**	0.26	0.9909	0.9930	0.9920	2.06
7		T	168,602	165,819	**167,211**	1.18	0.9995	0.9995	0.9995	0.83
8		S	188,198	191,709	**189,954**	1.31	0.9995	0.9997	0.9996	1.32
9		p-X	188,510	184,038	**186,274**	1.70	0.9997	0.9987	0.9992	0.49
10		m-X	197,068	193,888	**195,478**	1.15	0.9992	0.9994	0.9993	0.56
11		o-X	198,376	194,140	**196,258**	1.53	0.9991	0.9991	0.9991	0.73

12	Carboxylic	PPA	26,574	25,963	**26,269**	1.64	0.9977	0.9953	0.9965	1.68
13		BTA	71,259	67,832	**69,546**	3.48	0.9963	0.9967	0.9965	0.13
14		IVA	99,441	94,589	**97,015**	3.54	0.9965	0.9935	0.9950	2.09
15		VLA	79,615	78,949	**79,282**	0.59	0.9918	0.9925	0.9922	0.97

16	Ketone	MEK	48,980	48,566	**48,773**	0.60	0.9969	0.9987	0.9978	1.79
17		MIBK	117,383	117,646	**117,515**	0.16	0.9998	0.9985	0.9992	0.85

18	Alcohol	i-BuAl	93,667	92,778	**93,223**	0.67	0.9969	0.9972	0.9971	1.73

19	Ester	BuAc	121,114	117,791	**119,453**	1.97	0.9982	0.9973	0.9978	0.79

	Mean					1.30	0.9954	0.9954	0.9954	1.39
	SD					1.12	0.0085	0.0067	0.0075	0.82

^a^CV (coefficient of variation) = SD/mean ∗ 100.

^b^1 *μ*L injection of 4th calibration point (mean 21.6 ng *μ*L^−1^ F-WS) for five replicate analyses.

**Table 5:** Comparison of percent difference (PD) between the actual and projected response factor (RF) values for all (*n* = 18) or three chemical groups (*n* = 14) in relation to three major variables of reference VOCs used for prediction: (1) carbon number, (2) molecular weight, and (3) boiling point.

Order	Variables	Functional group	Number of chemicals	PD^a^		Predictive equation^b^		*R* ^2^	*P* value
				Mean	SD	Slope	Intercept		
1	Carbon number	All compounds^c^	18	17.9	19.0	34,175	−82,472	0.9396	3.58*E* − 11
2		Aldehyde	4	9.83	6.55	24,836	−60,290	0.9725	1.39*E* − 02
3		Aromatic	6	2.03	0.76	29,456	−43,139	0.9713	3.13*E* − 04
4		Carboxylic	4	12.7	4.30	29,818	−58,701	0.9012	5.07*E* − 02

	Mean							0.9462	0.0162
	SD							0.0336	0.0239

5	Molecular weight	All compounds	18	40.6	27.4	2,784	−145,760	0.5445	4.73*E* − 04
6		Aldehyde	4	9.79	6.49	1,773	−88,781	0.9726	1.38*E* − 02
7		Aromatic	6	1.73	1.22	2,142	−33,879	0.9738	2.60*E* − 04
8		Carboxylic	4	12.7	4.28	2,126	−126,763	0.9013	5.06*E* − 02

	Mean							0.8481	0.0163
	SD							0.2052	0.0238

9	Boiling point	All compounds	18	81.5	112	609	31,947	0.1404	1.26*E* − 01
10		Aldehyde	4	16.2	10.0	961	−30,600	0.8870	5.82*E* − 02
11		Aromatic	6	2.08	0.89	949	58,007	0.9687	3.71*E* − 04
12		Carboxylic	4	17.7	7.19	1,358	−158,432	0.7813	1.16*E* − 01

	Mean							0.6944	7.51*E* − 02
	SD							0.3772	5.80*E* − 02

^a^Percent difference (PD) = |(RF (projected) − RF (actual))|/RF(actual) ∗ 100.

^b^The predictive equations are derived from linear regression analysis between the number of carbon (*x*-axis) and actual RF values (*y*-axis).

^c^AA is not considered.

**Table 6:** Assessment of the PD values between the actual and projected RF values for arbitrarily divided chemical groups.

Order	Type of VOC groups	Number of chemical	Projected equation^a^		*R* ^2^	*P* value	PD values^b^ of all and six individual (nonmodified) groups^c^						
			Slope	Intercept			All	I	II	III	IV	V	VI
(A) 6 original functional groups													

1	All	18	34,175	−82,472	0.9396	3.58*E* − 11	17.9	43.6	3.52	16.5	7.75	41.8	2.62
2	Aldehyde (I)	4	24,836	−60,290	0.9725	1.39*E* − 02	9.83	9.83					
3	Aromatic (II)	6	29,456	−43,139	0.9713	3.13*E* − 04	2.03		2.03				
4	Carboxylic (III)	4	29,818	−58,701	0.9012	5.07*E* − 02	12.7			12.7			
5	Ketone (IV)^d^	2	∗	∗	∗	∗	∗	∗	∗	∗	∗	∗	∗
6	Alcohol (V)^d^	1	∗	∗	∗	∗	∗	∗	∗	∗	∗	∗	∗
7	Ester (VI)^d^	1	∗	∗	∗	∗	∗	∗	∗	∗	∗	∗	∗

(B) 25 arbitrary groups													

1	II + V	7	25,484	−12,686	0.9823	1.42*E* − 05	2.83	—^e^	2.60	—	—	4.26	—
2	II + VI	6	32,806	−69,569	0.9668	6.89*E* − 05	3.17	—	2.61	—	—	—	6.54
3	II + IV	8	35,411	−89,325	0.9856	9.35*E* − 07	3.97	—	3.29	—	6.03	—	—
4	II + III + VI	11	32,994	−71,393	0.9857	1.31*E* − 09	5.63	—	2.74	9.87	—	—	5.96
5	II + III	10	32,949	−70,402	0.9872	7.36*E* − 09	5.73	—	2.59	10.4	—	—	—
6	II + III + IV	12	33,650	−76,475	0.9845	2.18*E* − 10	7.33	—	2.94	11.1	13.0	—	—
7	II + III + V	11	31,181	−56,899	0.9654	6.96*E* − 08	9.89	—	2.11	17.2	—	27.2	—
8	III + VI	5	29,615	−57,914	0.9445	5.65*E* − 03	10.3	—	—	12.9	—	—	0.27
9	III + IV	6	30,661	−64,601	0.8746	1.96*E* − 02	12.0	—	—	10.3	19.0	—	—
10	I + IV	6	31,804	−85,229	0.9241	2.22*E* − 03	13.1	13.6	—	—	12.0	—	—
11	III + V	5	27,486	−42,376	0.6568	9.62*E* − 02	13.1	—	—	19.3	—	27.5	—
12	I + II + IV	12	37,696	−108,341	0.9787	1.09*E* − 09	13.1	29.7	4.26	—	6.63	—	—
13	I + II + III + IV	16	35,474	−91,942	0.9664	1.04*E* − 11	14.3	27.1	3.77	23.2	2.65	—	—
14	I + II + VI	11	38,121	−111,370	0.9773	1.05*E* − 08	14.7	33.4	4.32	—	—	—	1.75
15	I + II + III + VI	15	35,445	−91,569	0.9646	8.12*E* − 11	15.2	28.1	3.73	22.8	—	—	1.38
16	I + II	10	38,135	−111,661	0.9774	7.25*E* − 08	16.2	33.9	4.37	—	—	—	—
17	I + II + III	14	35,458	−91,524	0.9646	4.50*E* − 10	16.2	28.4	3.71	22.7	—	—	—
18	I + II + V	11	35,227	−89,454	0.9322	1.47*E* − 06	17.9	32.6	3.58	—	—	44.8	—
19	I + VI	5	33,109	−92,201	0.9272	8.52*E* − 03	18.7	20.6	—	—	—	—	10.9
20	I + II + III + V	15	34,080	−81,036	0.9372	3.43*E* − 09	20.7	47.4	3.34	15.1	—	40.7	—
21	I + III + IV	10	29,862	−69,448	0.8341	2.22*E* − 04	21.7	34.5	—	17.5	4.57	—	—
22	I + III + VI	9	30,162	−70,431	0.8311	6.19*E* − 04	24.1	34.9	—	17.5	—	—	7.46
23	I + III	8	27,327	−59,496	0.7476	5.59*E* − 03	26.8	37.1	—	16.5	—	—	—
24	I + III + V	9	25,591	−47,341	0.5444	2.32*E* − 02	35.8	57.4	—	12.9	—	41.0	—
25	I + V	5	20,967	−33,206	0.3434	2.99*E* − 01	47.7	48.3	—	—	—	45.7	—

Statistics	Mean				0.8955	0.0188	14.9	33.0	3.26	15.8	8.95	34.1	4.61
(*n* = 29)^d^	SD				0.1490	0.0586	9.90	12.1	0.74	4.45	5.36	14.0	3.67
	Min						2.03	9.83	2.03	9.87	2.65	4.26	0.27

	*N *						29	17	17	17	8	8	8

^a^The projected equations are derived from linear regression analysis between the number of carbon (*x*-axis) and actual RF values (*y*-axis).

^b^Percent difference (PD) = |(RF (Projected) − RF (Actual))|/RF(Actual) ∗ 100.

^c^I: aldehyde, II: aromatic, III: carboxylic, IV: ketone, V: alcohol, and VI: ester.

^d^As three groups (ketone (IV), alcohol (V), and ester (VI)) have only the limited number of components (less than 2), their predictive equations cannot be made and are not considered from counting of total group numbers (7(A) + 25(B) − 3(A) = 29).

^e^Not computed.

**Table 7:** Results of the best projected RF for individual compound when matching the best fit equation with each of the six original VOC functional groups representing all 18 reference compounds.

Order	Grouping code^a^	Compound	Carbon number	Actual RF	Projected RF^b^	PD^c^
1	Aldehyde (I)^d^	PA	3	11,984	14,217	**18.6 **
2		BA	4	43,520	39,053	**10.3 **
3		IA	5	65,981	63,888	**3.17 **
4		VA	5	59,563	63,888	**7.26 **

5	Aromatic (II)	B	6	131,520	133,598	**1.58 **
6		T	7	167,211	163,054	**2.49 **
7		S	8	189,954	192,510	**1.35 **
8		p-X	8	186,274	192,510	**3.35 **
9		m-X	8	195,478	192,510	**1.52 **
10		o-X	8	196,258	192,510	**1.91 **

11	Carboxylic (II + III + VI)	PPA	3	26,269	27,588	**5.02 **
12		BTA	4	69,546	60,582	**12.9 **
13		IVA	5	97,015	93,576	**3.54 **
14		VLA	5	79,282	93,576	**18.0 **

15	Ketone (I + II + III + IV)	MEK	4	48,773	49,955	**2.42 **
16		MIBK	6	117,515	120,904	**2.88 **

17	Alcohol (II + V)	i-BuAl	4	93,223	89,250	**4.26 **

18	Ester (III + VI)	BuAc	6	119,453	119,773	**0.27 **

	Mean					**5.60 **
	SD					**5.63 **

^a^Predictive equations ((1) slopes and (2) intercepts) developed for 29 arbitrary groups (codes) in Table 6 are used.

(1) Slope: Eqn (I) = 24,836, Eqn (II) = 29,456, Eqn (II + III + VI) = 32,994, Eqn (I + II + III + IV) = 35,474, Eqn (II + V) = 25,484, and Eqn (III +VI) = 29,615.

(2) Intercept: Eqn (I) = −60,290, Eqn (II) = −43,139, Eqn (II + III + VI) = −71,393, Eqn (I + II + III + IV)) = −91,942, Eqn (II + V) = −12,686, and Eqn (III** **+** **VI)** **=** **−57,914.

^b^The best projected RFs are derived by taking the minimum PD value for each compound (out of 18) after testing against 29 linear regression equations (between the number of carbon (*x*-axis) and actual RF values (*y*-axis)).

^c^Percent difference (PD) = |(RF (Projected) − RF (Actual))| × 100/RF (Actual): here, the AA data are excluded due to the eccentricity.

^d^Best fit equation (Roman letter) for a given chemical group is shown in the parenthesis.

## Abbreviations

AA: Acetaldehyde
B: Benzene
BA: Butyraldehyde
BP: Boiling point
BTA: Butyric acid
CN: Carbon number
CLASS: Compounds lacking authentic standards/surrogates
CV: Coefficients of variation
DI: Direct injection
DILS: Direct injection of liquid standard
FID: Flame ionization detector
GC: Gas chromatograph
HS-SPME: Headspace solid-phase micro extraction
IA: Isovaleraldehyde
I-BuAl: Isobutyl alcohol
IVA: Isovaleric acid
LR: Linear regression
L-WS: Liquid working standard
MDL: Method detection limit
MEK: Methyl ethyl ketone
MIBK: Methyl isobutyl ketone
MS: Mass spectrometer
MW: Molecular weight
m-X: *m*-Xylene
BuAc: n-Butyl acetate
o-X: *o*-Xylene
PA: Propionaldehyde
PD: Percent difference
PPA: Propionic acid
p-X: *p*-Xylene
RF: Response factor
RSE: Relative standard errors
S: Styrene
SPME: Solid-phase microextraction
ST: Sorbent tube
T: Toluene
TD: Thermal desorption
VA: n-Valeraldehyde
VLA: n-Valeric acid
VOC: Volatile organic compound.

## References

[1] Fenselau C (1974). Gas chromatography mass spectrometry: a report on the state of the art. *Applied Spectroscopy*.

[2] Demeestere K, Dewulf J, de Witte B, Van Langenhove H (2007). Sample preparation for the analysis of volatile organic compounds in air and water matrices. *Journal of Chromatography A*.

[3] Beran JA, Kevan L (1969). Molecular electron ionization cross sections at 70 eV. *Journal of Physical Chemistry*.

[4] Ahn JW, Pandey SK, Kim KH (2011). Comparison of GC-MS calibration properties of volatile organic compounds and relative quantification without calibration standards. *Journal of Chromatographic Science*.

[5] Kabir E, Kim KH (2012). Use of solid phase micro extraction (SPME) in the analysis of the reduced sulfur compounds (RSC) and its experimental limitations. *Microchemical Journal*.

[6] Woolfenden E (2010). Sorbent-based sampling methods for volatile and semi-volatile organic compounds in air, part 2: sorbent selection and other aspects of optimizing air monitoring methods. *Journal of Chromatography A*.

[7] Wu CH, Feng CT, Lo YS, Lin TY, Lo JG (2004). Determination of volatile organic compounds in workplace air by multisorbent adsorption/thermal desorption-GC/MS. *Chemosphere*.

[8] Pandey SK, Kim KH (2009). Simultaneous determination of odorous volatile organic compounds with gas chromatography and a thermal desorber: a case study on methyl ethyl ketone, methyl isobutyl ketone, butyl acetate, toluene, and xylene. *Microchemical Journal*.

[9] Parker DB, Gilley J, Woodbury B (2013). Odorous VOC emission following land application of swine manure slurry. *Atmospheric Environment*.

[10] Kim YH, Kim KH Novel approach to test the relative recovery of liquid-phase standard in sorbent-tube analysis of gaseous volatile organic compounds. *Analytical Chemistry*.

[11] Kim YH, Kim KH Validation of CLASS method by real samples with different target compounds.

[12] Allgood C, Orlando R, Munson B (1990). Correlations of relative sensitivities in gas chromatography electron ionization mass spectrometry with molecular parameters. *Journal of the American Society for Mass Spectrometry*.

