# Supplementary material for: A Statistical Estimation Approach for Quantitative Concentrations of Compounds Lacking Authentic Standards/Surrogates Based on Linear Correlations between Directly Measured Detector Responses and Carbon Number of Different Functional Groups
Source: ScientificWorldJournal. 2013 May 12;2013:241585. doi: 10.1155/2013/241585 (PMC3671284; doi:10.1155/2013/241585)
Supplement: Supplementary file 1 — Table 1S: Preparation of liquid phase standard of 19 VOC for the analysis by the TD-GC-MS based analysis in this study. Table 2S: List of the 54 VOCs investigated in previous study (Exp-DI and -SPME) by Ahn et al. [4]. Table 3S: Results of the projected RF values derived by simplification of projected grouping. Table 4S: Operational conditions of GC-MS system for the analysis of reference VOCs in previous study of Ahn et al. [4]. Table 5S: Comparison of percent difference (PD) between actual and predicted response factor (RF) values for all (n=49) or two chemical groups (n=41) in relation to three major variables of reference VOCs used for prediction: (1) carbon number, (2) molecular weight, and (3) boiling point (Ahn et al. [4]). Table 6S: Assessment of the least PD values between the actual and projected RF values for arbitrarily divided chemical groups based on linear regression analysis using Exp-DI and-SPME data (Ahn et al. [4]). Table 7S: Results of the minimum percent difference (PD) values between the best projected and actual RF in the study of Ahn et al. [4]. Fig. 1S: The correlations between RF values and key variables (A. carbon number, B. molecular weight, and C. boiling point) in Ahn et al. [4]. [file 241585.f1.docx]

| Table 1S. Preparation of liquid phase standard of 19 VOC for the analysis by the TD-GC-MS based analysis in this study. | | | | | | | | | | | | | | | | | | | | | | | | | | | | | | | | | | | | | | | | | | |
| --- | --- | --- | --- | --- | --- | --- | --- | --- | --- | --- | --- | --- | --- | --- | --- | --- | --- | --- | --- | --- | --- | --- | --- | --- | --- | --- | --- | --- | --- | --- | --- | --- | --- | --- | --- | --- | --- | --- | --- | --- | --- | --- |
| A. Preparation of liquid phase standard for 19 VOCs | | | | | | | | | | |  | |  | |  | |  | |  | |  | |  | |  | |  | |  | |  | |  | |  | |  | |  | |  | |
|  | Compound^s^ | | Methanol | | AA | | PA | | BA | | IA | | VA | | B | | T | | S | | p-X | | m-X | | o-X | | MEK | | MIBK | | i-BuAl | | BuAc | | PPA | | BTA | | IVA | | VLA | |
| **Primary grade chemical** | Concentration (%) | |  | | 99.0 | | 97.0 | | 99.0 | | 97.0 | | 97.0 | | 99.5 | | 99.5 | | 99.0 | | 99.0 | | 99.0 | | 97.0 | | 99.0 | | 99.5 | | 99.0 | | 99.5 | | 99.0 | | 99.0 | | 99.0 | | 99.0 | |
| **PS^a^** | Volume (μL) | | 13,700 | | 900 | | 300 | | 300 | | 300 | | 300 | | 300 | | 300 | | 300 | | 300 | | 300 | | 300 | | 300 | | 300 | | 300 | | 300 | | 300 | | 300 | | 300 | | 300 | |
|  | Concentration (ng μL^-1^) | |  | | 34,972 | | 11,611 | | 11,954 | | 11,596 | | 11,786 | | 13,104 | | 12,925 | | 13,454 | | 12,845 | | 12,845 | | 12,804 | | 11,954 | | 11,970 | | 11,895 | | 13,149 | | 14,702 | | 14,226 | | 13,736 | | 13,929 | |
| **1st L-WS^b^** | volume (mL) | | 19,800 | | 200 | | 200 | | 200 | | 200 | | 200 | | 200 | | 200 | | 200 | | 200 | | 200 | | 200 | | 200 | | 200 | | 200 | | 200 | | 200 | | 200 | | 200 | | 200 | |
|  | Concentration (ng μL^-1^) | |  | | 350 | | 116 | | 120 | | 116 | | 118 | | 131 | | 129 | | 135 | | 128 | | 128 | | 128 | | 120 | | 120 | | 119 | | 131 | | 147 | | 142 | | 137 | | 139 | |
| ^a^ PS: Dilution of pure chemical (primary grade chemical) to make 20 mL solution. | | | | | | | | | | | | |  | |  | |  | |  | |  | |  | |  | |  | |  | |  | |  | |  | |  | |  | |  | |
| ^b^ 1st L-WS: Dilution of PS to make in 20 mL solution. | | | | | | | | | | |  | |  | |  | |  | |  | |  | |  | |  | |  | |  | |  | |  | |  | |  | |  | |  | |
|  |  |  | |  | |  | |  | |  | |  | |  | |  | |  | |  | |  | |  | |  | |  | |  | |  | |  | |  | |  | |  | |  |
| B. Preparation of F-WS for 5 point calibration: absolute mass (ng) of VOC loaded on each tube sampler. | | | | | | | | | | | | | | | | | | |  | |  | |  | |  | |  | |  | |  | |  | |  | |  | |  | |  | |
| Order | Mixing volume (μL) | | | | Concentration^c^ (ng μL^-1^) | | | | | | | | | |  | |  | |  | |  | |  | |  | |  | |  | |  | |  | |  | |  | |  | |  | |
|  | 1st L-WS | | Methanol | | AA | | PA | | BA | | IA | | VA | | B | | T | | S | | p-X | | m-X | | o-X | | MEK | | MIBK | | i-BuAl | | BuAc | | PPA | | BTA | | IVA | | VLA | |
| 1 | 14 | | 1,486 | | 3.26 | | 1.08 | | 1.12 | | 1.08 | | 1.10 | | 1.22 | | 1.21 | | 1.26 | | 1.20 | | 1.20 | | 1.20 | | 1.12 | | 1.12 | | 1.11 | | 1.23 | | 1.37 | | 1.33 | | 1.28 | | 1.30 | |
| 2 | 70 | | 1,430 | | 16.3 | | 5.42 | | 5.58 | | 5.41 | | 5.50 | | 6.12 | | 6.03 | | 6.28 | | 5.99 | | 5.99 | | 5.98 | | 5.58 | | 5.59 | | 5.55 | | 6.14 | | 6.86 | | 6.64 | | 6.41 | | 6.50 | |
| 3 | 140 | | 1,360 | | 32.6 | | 10.8 | | 11.2 | | 10.8 | | 11.0 | | 12.2 | | 12.1 | | 12.6 | | 12.0 | | 12.0 | | 12.0 | | 11.2 | | 11.2 | | 11.1 | | 12.3 | | 13.7 | | 13.3 | | 12.8 | | 13.0 | |
| 4 | 280 | | 1,220 | | 65.3 | | 21.7 | | 22.3 | | 21.6 | | 22.0 | | 24.5 | | 24.1 | | 25.1 | | 24.0 | | 24.0 | | 23.9 | | 22.3 | | 22.3 | | 22.2 | | 24.5 | | 27.4 | | 26.6 | | 25.6 | | 26.0 | |
| 5 | 700 | | 800 | | 163 | | 54.2 | | 55.8 | | 54.1 | | 55.0 | | 61.2 | | 60.3 | | 62.8 | | 59.9 | | 59.9 | | 59.8 | | 55.8 | | 55.9 | | 55.5 | | 61.4 | | 68.6 | | 66.4 | | 64.1 | | 65.0 | |

| Table 2S. List of the 54 VOCs investigated in previous study (Exp-DI and -SPME) by Ahn et al. [4]. | | | | | | |  |
| --- | --- | --- | --- | --- | --- | --- | --- |
|  |  |  |  |  |  |  |  |
| Order | VOC | Compounds^a^ | Carbon | MW | Boiling point | Formula | CAS |
|  | Groups |  | numbers | (g mol^-1^) | (℃) |  | number |
|  |  |  |  |  |  |  |  |
| 1 |  | Chloroform | 1 | 118 | 61.2 | CHCl3 | 67-66-3 |
| 2 |  | Bromochloromethane | 1 | 128 | 68.1 | CH2BrCl | 74-97-5 |
| 3 |  | Carbon tetrachloride | 1 | 152 | 76.72 | CCl4 | 56-23-5 |
| 4 |  | Bromodichloromethane | 1 | 162 | 90 | CHBrCl2 | 75-27-4 |
| 5 |  | Dibromomethane | 1 | 172 | 96-98 | CH2Br2 | 74-95-3 |
| 6 |  | Dibromochloromethane | 1 | 206 | 119-120 | CHBr2Cl | 124-48-1 |
| 7 | Haloalkane | Bromoform | 1 | 250 | 147-151 | CHCBr3 | 75-25-2 |
| 8 |  | 1,1-Dichloroethane | 2 | 98 | 57.2 | C2H4Cl2 | 75-34-3 |
| 9 |  | 1,1,1-Trichloroethane | 2 | 132 | 74 | C2H3Cl3 | 71-55-6 |
| 10 |  | 1,1,2-Trichloroethane | 2 | 132 | 110-115 | C2H3Cl3 | 79-00-5 |
| 11 |  | 1,2-Dibromoethane | 2 | 186 | 129-133 | C2H4Br2 | 106-93-4 |
| 12 |  | 1,1,2,2-Tetrachloroethane | 2 | 166 | 146.5 | C2H2Cl4 | 79-34-5 |
| 13 |  | 2,2-Dichloropropane | 3 | 112 | 68-69 | C3H6Cl2 | 594-20-7 |
| 14 |  | 1,2-Dichloropropane | 3 | 112 | 95-96 | C3H6Cl2 | 78-87-5 |
| 15 |  | 1,3-Dichloropropane | 3 | 112 | 122 | C3H6Cl2 | 142-28-9 |
| 16 |  | 1,2,3-Trichloropropane | 3 | 146 | 156.85 | C3H5Cl3 | 96-18-4 |
| 17 |  | 1,2-Dibromo-3-chloropropane | 3 | 234 | 195 | C3H5Br2Cl | 96-12-8 |
| 18 |  | 1,2-Dichloroethane | 2 | 98 | 84 | C2H4Cl2 | 107-06-2 |
| 19 |  | 1,1,1,2-Tetrachloroethane | 2 | 168 | 130.5 | C2H2Cl4 | 630-20-6 |
| 20 |  | 1,1-Dichloropropene | 3 | 110 | 76-77 | C3H4Cl2 | 563-58-6 |
| 21 | Chloropropene | cis-1,3-Dichloropropene | 3 | 110 | 104 | C3H4Cl2 | 10061-01-5 |
| 22 |  | trans-1,3-Dichloropropene | 3 | 110 | 112 | C3H4Cl2 | 10061-02-6 |
| 23 |  | 1,1-Dichloroethylene | 2 | 96 | 32 | C2H2Cl2 | 75-35-4 |
| 24 |  | trans-1,2-Dichloroethylene | 2 | 96 | 47.5 | C2H2Cl2 | 156-60-5 |
| 25 | Chloroethene | cis-1,2-Dichloroethylene | 2 | 96 | 60.3 | C2H2Cl2 | 156-59-2 |
| 26 |  | Trichloroethylene | 2 | 130 | 87.2 | C2HCl3 | 79-01-6 |
| 27 |  | Tetrachloroethylene | 2 | 164 | 121.1 | C2Cl4 | 127-18-4 |
| 28 |  | Methylene chloride | 1 | 84 | 39.6 | CH2Cl2 | 75-09-2 |
| 29 |  | Benzene | 6 | 78 | 80.1 | C6H6 | 71-43-2 |
| 30 |  | Chlorobenzene | 6 | 112 | 131 | C6H5Cl | 108-90-7 |
| 31 |  | Bromobenzene | 6 | 156 | 156 | C6H5Br | 108-86-1 |
| 32 |  | 1,3-Dichlorobenzene | 6 | 146 | 172-173 | C6H4Cl2 | 541-73-1 |
| 33 |  | 1,4-Dichlorobenzene | 6 | 146 | 174 | C6H4Cl2 | 106-46-7 |
| 34 |  | 1,2-Dichlorobenzene | 6 | 146 | 180.5 | C6H4Cl2 | 95-50-1 |
| 35 |  | 1,2,4-Trichlorobenzene | 6 | 180 | 214.4 | C6H3Cl3 | 120-82-1 |
| 36 |  | 1,2,3-Trichlorobenzene | 6 | 180 | 218-219 | C6H3Cl3 | 87-61-6 |
| 37 | Aromatic | Toluene | 7 | 92 | 110.6 | C7H8 | 108-88-3 |
| 38 |  | 2-Chlorotoluene | 7 | 126 | 159 | C7H7Cl | 95-49-8 |
| 39 |  | 4-Chlorotoluene | 7 | 126 | 162 | C7H7Cl | 106-43-4 |
| 40 |  | Ethylbenzene | 8 | 106 | 136 | C8H10 | 100-41-4 |
| 41 |  | m-Xylene | 8 | 106 | 139 | C8H10 | 108-38-3 |
| 42 |  | o-Xylene | 8 | 106 | 144.4 | C8H10 | 95-47-6 |
| 43 |  | Styrene | 8 | 104 | 145 | C8H8 | 100-42-5 |
| 44 |  | Isopropylbenzene | 9 | 120 | 152 | C9H12 | 98-82-8 |
| 45 |  | n-Propylbenzene | 9 | 120 | 158 | C9H12 | 103-65-1 |
| 46 |  | 1,3,5-Trimethylbenzene | 9 | 120 | 164.7 | C9H12 | 108-67-8 |
| 47 |  | 1,2,4-Trimethylbenzene | 9 | 120 | 169-171 | C9H12 | 95-63-6 |
| 48 |  | tert-Butylbenzene | 10 | 134 | 169 | C10H14 | 98-06-6 |
| 49 |  | sec-Butylbenzene | 10 | 134 | 173-174 | C10H14 | 135-98-8 |
| 50 |  | p-Isopropyltoluene | 10 | 134 | 176-178 | C10H14 | 99-87-6 |
| 51 |  | n-Butylbenzene | 10 | 134 | 183 | C10H14 | 104-51-8 |
| 52 |  | Naphtalene | 10 | 128 | 218 | C10H8 | 91-20-3 |
| 53 |  | p-Xylene | 8 | 106 | 138.35 | C8H10 | 106-42-3 |
| 54 | Diene | Hexachlorobutadiene | 4 | 258 | 210-220 | C4Cl6 | 87-68-3 |
| ^a^ 49 compounds except for five compounds (1,2-dichloroethane, 1,1,1,2-tetrachloroethane, methylene chloride, p-xylene, and hexachlorobutadiene) are used to calculate the projected equation for response factor. | | | | | | | |

| Table 3S. Results of the projected RF values derived by simplification of projected grouping. | | | | | | |
| --- | --- | --- | --- | --- | --- | --- |
| Order | Group | Compound | Carbon | Actual | Projected | **PD ^c^** |
|  |  |  | number | RF | RF ^b^ |  |
| **A. 1st group** ^a^ | |  |  |  |  |  |
| 1 |  | PA | 3 | 11,984 | 10,183 | **17.7** |
| 2 | Aldehyde | BA | 4 | 43,520 | 41,987 | **3.65** |
| 3 |  | IA | 5 | 65,981 | 73,791 | **10.6** |
| 4 |  | VA | 5 | 59,563 | 73,791 | **19.3** |
| 5 | Ketone | MEK | 4 | 48,773 | 41,987 | **16.2** |
| 6 |  | MIBK | 6 | 117,515 | 105,595 | **11.3** |
|  |  |  |  |  |  |  |
| **B. 2nd group** ^a^ | |  |  |  |  |  |
| 7 |  | B | 6 | 131,520 | 136,554 | **3.69** |
| 8 |  | T | 7 | 167,211 | 163,259 | **2.42** |
| 9 | Aromatic | S | 8 | 189,954 | 189,965 | **0.01** |
| 10 |  | p-X | 8 | 186,274 | 189,965 | **1.94** |
| 11 |  | m-X | 8 | 195,478 | 189,965 | **2.90** |
| 12 |  | o-X | 8 | 196,258 | 189,965 | **3.31** |
| 13 | Alcohol | i-BuAl | 4 | 93,223 | 83,143 | **12.1** |
| 14 | Ester | BuAc | 6 | 119,453 | 136,554 | **12.5** |
|  |  |  |  |  |  |  |
| **C. 3rd group** ^a^ | |  |  |  |  |  |
| 15 |  | PPA | 3 | 26,269 | 30,755 | **14.6** |
| 16 | Carboxylic | BTA | 4 | 69,546 | 60,573 | **14.8** |
| 17 |  | IVA | 5 | 97,015 | 90,392 | **7.33** |
| 18 |  | VLA | 5 | 79,282 | 90,392 | **12.3** |
|  | Mean |  |  |  |  | **9.25** |
|  | SD |  |  |  |  | **6.14** |
| ^a^ (1) Slopes and (2) intercepts yielded by each group. | | | |  |  |  |
| A. 1st group: (1) slope = 31,804 and (2) intercept = -85,229 | | | | |  |  |
| B. 2nd group: (1) slope = 26,706 and (2) intercept = -23,679 | | | | |  |  |
| C. 3rd group: (1) slope = 29,818 and (2) intercept = -58,701 | | | | |  |  |
| ^b^The projected RFs are derived from linear regression analysis between the number of carbon (X axis) and actual RF values (Y axis). | | | | | | |
| ^c^ Percent difference (PD) = \|(RF(Projected) - RF (Actual)\| / RF(Actual) * 100. | | | | | |  |

| Table 4S. Operational conditions of GC-MS system for the analysis of reference VOCs in previous study of Ahn et al. [4]. | | | | | | | | |
| --- | --- | --- | --- | --- | --- | --- | --- | --- |
| GC (Shimadzu GC-2010, JAPAN) and MS (Shimadzu GCMS-QP2010, JAPAN) | | | | | | |  |  |
| Column: Vocol (diameter: 0.32 mm, length: 60 m, and film thickness: 1.8 µm, Supelco) | | | | | | |  |  |
| Injector setting |  |  | Detector setting | | |  |  |  |
| Temp. | 250 ℃ |  | Ionization mode: | | | EI (70 eV) |  |  |
| Carrier gas: | He (99.999%) |  | Ion source temp.: | | | 200 ℃ |  |  |
| Column flow: | 1.2 mL min^-1^ |  | Interface temp.: | | | 200 ℃ |  |  |
| Split flow: | 12 mL min^-1^ |  | TIC scan range: | | | 35~250 m z^-1^ |  |  |
| Split ratio | 1:10 |  |  |  |  |  |  |  |
|  |  |  |  | | |  |  |  |
|  |  |  |  | | |  |  |  |
| Oven setting |  |  | SPME setting | | |  |  |  |
| Oven temp: | 35 ℃ (4 min) |  | Fiber: CAR-PDMS, 75 µm, Supelco | | | |  |  |
| Oven rate: | 4 ℃ min^-1^ |  | Conditioning: 300 ℃ for 30 min | | | |  |  |
| Max oven temp: | 200 ℃ (10 min) |  | Sample absorption time: 30 min | | | |  |  |
| Total time: | 41.25 min |  | Sample desorption time: 5 min | | | |  |  |
| Carrier gas: | He (99.999%) |  |  | | |  |  |  |
| Carrier gas flow: | 1 mL min^-1^ |  |  | | |  |  |  |
|  |  |  |  | | |  |  |  |

| Table 5S. Comparison of percent difference (PD) between actual and predicted response factor (RF) values for all (n=49) or two chemical groups (n=41) in relation to three major variables of reference VOCs used for prediction: (1) carbon number, (2) molecular weight, and (3) boiling point (Ahn et al. [4]). | | | | | | | | | | | |
| --- | --- | --- | --- | --- | --- | --- | --- | --- | --- | --- | --- |
|  | | | | | | | | | |  |  |
| Order | Variables | Functional | Number |  | PD^a^ | |  | Projected equation | | R^2^ | P-value |
|  |  | group |  |  | Mean | SD |  | Slope | Intercept |  |  |
|  |  |  |  |  |  |  |  |  |  |  |  |
| **A. Exp-DI** | |  |  |  |  |  |  |  |  |  |  |
| 1 | Carbon | All compounds^b^ | 49 |  | 31.3 | 38.7 |  | 52,715 | 30,661 | 0.7430 | 1.82E-15 |
| 2 | Number | Haloalkane | 17 |  | 30.4 | 30.4 |  | 27,880 | 81,884 | 0.1654 | 1.05E-01 |
| 3 |  | Aromatic | 24 |  | 28.3 | 41.2 |  | 50,426 | 52,067 | 0.2726 | 8.87E-03 |
|  | Mean |  |  |  |  |  |  |  |  | 0.3937 | 3.80E-02 |
|  | SD |  |  |  |  |  |  |  |  | 0.3072 | 5.84E-02 |
| 4 | Molecular | All compounds | 49 |  | 89.5 | 83.0 |  | -681 | 378,243 | 0.0155 | 3.95E-01 |
| 5 | weight | Haloalkane | 17 |  | 35.9 | 17.2 |  | 166 | 108,823 | 0.0156 | 6.33E-01 |
| 6 |  | Aromatic | 24 |  | 31.2 | 56.0 |  | 685 | 357,694 | 0.0117 | 6.15E-01 |
|  | Mean |  |  |  |  |  |  |  |  | 0.0143 | 5.47E-01 |
|  | SD |  |  |  |  |  |  |  |  | 0.0022 | 1.33E-01 |
| 7 | Boiling | All compounds | 49 |  | 52.8 | 70.9 |  | 2,854 | -83,105 | 0.4922 | 1.96E-08 |
| 8 | point | Haloalkane | 17 |  | 30.2 | 23.4 |  | 899 | 38,037 | 0.3606 | 1.08E-02 |
| 9 |  | Aromatic | 24 |  | 29.6 | 46.8 |  | 1,885 | 139,543 | 0.1503 | 6.12E-02 |
|  | Mean |  |  |  |  |  |  |  |  | 0.3344 | 2.40E-02 |
|  | SD |  |  |  |  |  |  |  |  | 0.1725 | 3.27E-02 |
|  |  |  |  |  |  |  |  |  |  |  |  |
| **B. Exp-SPME** | |  |  |  |  |  |  |  |  |  |  |
| 1 | Carbon | All compounds | 49 |  | 44.0 | 95.3 |  | 23,642 | -11,192 | 0.8163 | 6.47E-19 |
| 2 | Number | Haloalkane | 17 |  | 50.0 | 61.2 |  | 6,309 | 11,738 | 0.1982 | 7.33E-02 |
| 3 |  | Aromatic | 24 |  | 11.8 | 13.3 |  | 16,711 | 47,219 | 0.2656 | 9.96E-03 |
|  | Mean |  |  |  |  |  |  |  |  | 0.4267 | 2.78E-02 |
|  | SD |  |  |  |  |  |  |  |  | 0.3391 | 3.98E-02 |
| 4 | Molecular | All compounds | 49 |  | 182 | 263 |  | -658 | 192,362 | 0.0794 | 4.99E-02 |
| 5 | weight | Haloalkane | 17 |  | 50.5 | 58.5 |  | -71,989 | 34,699 | 0.0687 | 3.10E-01 |
| 6 |  | Aromatic | 24 |  | 18.4 | 16.9 |  | -709 | 267,745 | 0.1114 | 1.11E-01 |
|  | Mean |  |  |  |  |  |  |  |  | 0.0865 | 1.57E-01 |
|  | SD |  |  |  |  |  |  |  |  | 0.0222 | 1.36E-01 |
| 7 | Boiling | All compounds | 49 |  | 127 | 196 |  | 1,008 | -26,936 | 0.3352 | 1.31E-05 |
| 8 | point | Haloalkane | 17 |  | 48.7 | 39.2 |  | 102 | 12,715 | 0.1080 | 1.98E-01 |
| 9 |  | Aromatic | 24 |  | 20.3 | 17.0 |  | -226 | 214,032 | 0.0192 | 5.19E-01 |
|  | Mean |  |  |  |  |  |  |  |  | 0.1541 | 2.39E-01 |
|  | SD |  |  |  |  |  |  |  |  | 0.1630 | 2.62E-01 |
| ^a^Percent difference (PD) = **\|**(RF(Projected) - RF (Actual) **\|** /RF(Actual) * 100.  ^b^ Five compounds of 1,2-dichloroethane, 1,1,1,2-tetrachloroethane, methylene chloride, p-xylene, and hexachlorobutadiene are not used. | | | | | | | | | | | |

| Table 6S. Assessment of the least PD values between the actual and projected RF values for arbitrarily divided chemical groups based on linear regression analysis using Exp-DI and-SPME data (Ahn et al. [4]). | | | | | | | | | | | | | | |
| --- | --- | --- | --- | --- | --- | --- | --- | --- | --- | --- | --- | --- | --- | --- |
| Order | Type of |  |  | Projected equation^a^ | |  | R^2^ | P-value |  | PD values^b^ of all and four individual group ^c^ | | | | |
|  | VOC groups | Number |  | Slope | Intercept |  |  |  |  | All | I | II | III | IV |
|  |  |  |  |  |  |  |  |  |  |  |  |  |  |  |
| **A. Exp-DI** | |  |  |  |  |  |  |  |  |  |  |  |  |  |
| a. 5 original functional groups | | |  |  |  |  |  |  |  |  |  |  |  |  |
| 1 | All | 49 |  | 52,715 | 30,661 |  | 0.7430 | 1.82E-15 |  | 31.3 | 35.5 | 13.0 | 43.7 | 28.0 |
| 2 | Haloalkane (I) | 17 |  | 27,880 | 81,884 |  | 0.1654 | 1.05E-01 |  | 30.4 | 30.4 |  |  |  |
| 3 | Chloropropene (II) | 3 |  | * | * |  | * | * |  | * | * | * | * | * |
| 4 | Chloroethene (III) | 5 |  | * | * |  | * | * |  | * | * | * | * | * |
| 5 | Aromatic (IV) | 24 |  | 50,426 | 52,067 |  | 0.2726 | 8.87E-03 |  | 28.3 |  |  |  | 28.3 |
|  |  |  |  |  |  |  |  |  |  |  |  |  |  |  |
| b. 10 arbitrary groups | |  |  |  |  |  |  |  |  |  |  |  |  |  |
| 1 | II + III | 8 |  | 51,931 | 12,313 |  | 0.3705 | 1.09E-01 |  | 24.2 |  | 7.20 | 34.4 |  |
| 2 | II + IV | 27 |  | 54,204 | 20,727 |  | 0.4697 | 8.00E-05 |  | 26.1 |  | 9.73 |  | 28.1 |
| 3 | I + II | 20 |  | 28,372 | 81,262 |  | 0.2051 | 4.50E-02 |  | 27.1 | 30.6 | 7.47 |  |  |
| 4 | II + III + IV | 32 |  | 55,423 | 10,855 |  | 0.6421 | 3.59E-08 |  | 27.6 |  | 7.38 | 37.0 | 28.1 |
| 5 | I + II + III | 25 |  | 28,664 | 76,301 |  | 0.1783 | 3.55E-02 |  | 28.9 | 28.6 | 8.1 | 42.6 |  |
| 6 | I + II + IV | 44 |  | 51,983 | 36,720 |  | 0.7225 | 2.91E-13 |  | 29.6 | 34.3 | 15.3 |  | 28.1 |
| 7 | III + IV | 29 |  | 54,901 | 15,330 |  | 0.5954 | 9.56E-07 |  | 29.9 |  |  | 38.6 | 28.1 |
| 8 | I + IV | 41 |  | 51,569 | 40,726 |  | 0.7131 | 3.98E-12 |  | 30.5 | 34.0 |  |  | 28.1 |
| 9 | I + III | 22 |  | 27,054 | 78,582 |  | 0.1338 | 9.41E-02 |  | 31.4 | 28.3 |  | 42.1 |  |
| 10 | I + III + IV | 46 |  | 52,452 | 33,321 |  | 0.7374 | 2.33E-14 |  | 32.5 | 35.0 |  | 45.1 | 28.1 |
| Statistics ^d^ | Mean |  |  |  |  |  | 0.4338 | 0.0332 |  | 28.9 | 31.6 | 9.20 | 40.0 | 28.1 |
| (n = 13) | SD |  |  |  |  |  | 0.2401 | 0.0448 |  | 2.35 | 2.81 | 3.14 | 3.99 | 0.08 |
|  | Min  N |  |  |  |  |  |  |  |  | 24.2  13 | 28.3  8 | 7.20  7 | 34.4  7 | 28.1  8 |
|  |  |  |  |  |  |  |  |  |  |  |  |  |  |  |
|  |  |  |  |  |  |  |  |  |  |  |  |  |  |  |
| **B. Exp-SPME** | |  |  |  |  |  |  |  |  |  |  |  |  |  |
| a. 5 original functional groups | | |  |  |  |  |  |  |  |  |  |  |  |  |
| 1 | All | 49 |  | 23,642 | -11,192 |  | 0.8163 | 6.47E-19 |  | 44.0 | 94.3 | 25.0 | 39.0 | 11.8 |
| 2 | Haloalkane (I) | 17 |  | 6,309 | 11,738 |  | 0.1982 | 7.33E-02 |  | 50.0 | 50.0 |  |  |  |
| 3 | Chloropropene (II) | 3 |  | * | * |  | * | * |  | * | * | * | * | * |
| 4 | Chloroethene (III) | 5 |  | * | * |  | * | * |  | * | * | * | * | * |
| 5 | Aromatic (IV) | 24 |  | 16,711 | 47,219 |  | 0.2656 | 9.96E-03 |  | 11.8 |  |  |  | 11.8 |
|  |  |  |  |  |  |  |  |  |  |  |  |  |  |  |
| b. 10 arbitrary groups | |  |  |  |  |  |  |  |  |  |  |  |  |  |
| 1 | II + IV | 27 |  | 21,692 | 5,903 |  | 0.5398 | 1.28E-05 |  | 15.0 |  | 43.9 |  | 11.4 |
| 2 | III + IV | 29 |  | 20,053 | 19,792 |  | 0.4259 | 1.24E-04 |  | 17.4 |  |  | 46.3 | 11.4 |
| 3 | II + III + IV | 32 |  | 21,323 | 8,892 |  | 0.6778 | 7.23E-09 |  | 21.3 |  | 47.7 | 53.2 | 11.4 |
| 4 | II + III | 8 |  | -2,312 | 57,834 |  | 0.0030 | 8.98E-01 |  | 40.9 |  | 16.4 | 55.6 |  |
| 5 | I + III + IV | 46 |  | 23,530 | -10,056 |  | 0.8127 | 1.31E-17 |  | 45.5 | 94.9 |  | 39.5 | 11.8 |
| 6 | I + IV | 41 |  | 24,179 | -15,499 |  | 0.8165 | 6.14E-16 |  | 46.0 | 93.8 |  |  | 12.0 |
| 7 | I + II | 20 |  | 10,166 | 6,865 |  | 0.3420 | 6.76E-03 |  | 51.3 | 56.0 | 24.3 |  |  |
| 8 | I + II + III | 25 |  | 9,818 | 12,777 |  | 0.1408 | 6.46E-02 |  | 58.0 | 70.9 | 15.8 | 39.5 |  |
| 9 | I + III | 22 |  | 7,418 | 16,177 |  | 0.0733 | 2.23E-01 |  | 61.3 | 67.9 |  | 39.1 |  |
| 10 | I + II + IV | 44 |  | 24,272 | 16,399 |  | 0.8220 | 2.44E-17 |  | 106 | 226 | 80.9 |  | 23.9 |
| Statistics ^d^ | Mean |  |  |  |  |  | 0.4265 | 0.1063 |  | 43.7 | 94.3 | 38.2 | 45.5 | 13.4 |
| (n = 13) | SD |  |  |  |  |  | 0.3022 | 0.2577 |  | 26.2 | 60.7 | 25.0 | 7.40 | 4.65 |
|  | Min  N |  |  |  |  |  |  |  |  | 11.8  13 | 50.0  8 | 15.8  7 | 39.1  7 | 11.4  8 |
| ^a^The projected equations are derived from linear regression analysis between the actual RF values (Y axis) and number of carbon (X axis). | | | | | | | | | | | | | |  |
| ^b^PD = \|(RF(Projected) - RF (Actual) **\|** /RF(Actual) * 100.  ^c^I = Haloalkane, II = Chloropropene, III = Chloroethene, and IV = Aromatic. | | | | | | | | |  |  |  |  |  |  |
| ^d^ 13 groups except for Chloropropene (II) and Chloroethene (III) were used.  * Results are excluded because of limited number of compounds with different carbon number for regression analysis. | | | | | | | | | | | | |  |  |

| Table 7S. Results of the minimum percent difference (PD) values between the best projected and actual RF in the study of Ahn et al. [4]. | | | | | | | | | | | |
| --- | --- | --- | --- | --- | --- | --- | --- | --- | --- | --- | --- |
| Order | Grouping | Compound | Carbon |  | **Exp-DI** | | |  | **Exp-SPME** | | |
|  | code ^a^ |  | number |  | Actual | Projected | **PD ^b^** |  | Actual | Projected | **PD** |
|  |  |  |  |  | RF | RF ^a^ |  |  | RF | RF |  |
|  |  |  |  |  |  |  |  |  |  |  |  |
| 1 |  | Chloroform | 1 |  | 125,753 | 105,636 | **16.0** |  | 23,671 | 18,047 | **23.8** |
| 2 |  | Bromochloromethane | 1 |  | 103,298 | 105,636 | **2.26** |  | 9,442 | 18,047 | **91.1** |
| 3 |  | Carbon tetrachloride | 1 |  | 97,155 | 105,636 | **8.73** |  | 17,798 | 18,047 | **1.40** |
| 4 |  | Bromodichloromethane | 1 |  | 98,572 | 105,636 | **7.17** |  | 22,456 | 18,047 | **19.6** |
| 5 |  | Dibromomethane | 1 |  | 89,562 | 105,636 | **17.9** |  | 14,524 | 18,047 | **24.3** |
| 6 |  | Dibromochloromethane | 1 |  | 112,445 | 105,636 | **6.06** |  | 20,428 | 18,047 | **11.7** |
| 7 | Haloalkane | Bromoform | 1 |  | 91,925 | 105,636 | **14.9** |  | 14,163 | 18,047 | **27.4** |
| 8 | (Exp-DI: I + III | 1,1-Dichloroethane | 2 |  | 105,231 | 132,690 | **26.1** |  | 14,482 | 24,355 | **68.2** |
| 9 | Exp-SPME: I) ^d^ | 1,1,1-Trichloroethane | 2 |  | 115,916 | 132,690 | **14.5** |  | 17,592 | 24,355 | **38.4** |
| 10 |  | 1,1,2-Trichloroethane | 2 |  | 200,720 | 132,690 | **33.9** |  | 38,314 | 24,355 | **36.4** |
| 11 |  | 1,2-Dibromoethane | 2 |  | 101,985 | 132,690 | **30.1** |  | 19,350 | 24,355 | **25.9** |
| 12 |  | 1,1,2,2-Tetrachloroethane | 2 |  | 263,647 | 132,690 | **49.7** |  | 39,725 | 24,355 | **38.7** |
| 13 |  | 2,2-Dichloropropane | 3 |  | 76,970 | 159,744 | **108** |  | 8,409 | 30,664 | **265** |
| 14 |  | 1,2-Dichloropropane | 3 |  | 159,566 | 159,744 | **0.11** |  | 39,323 | 30,664 | **22.0** |
| 15 |  | 1,3-Dichloropropane | 3 |  | 85,888 | 159,744 | **86.0** |  | 46,308 | 30,664 | **33.8** |
| 16 |  | 1,2,3-Trichloropropane | 3 |  | 237,992 | 159,744 | **32.9** |  | 40,046 | 30,664 | **23.4** |
| 17 |  | 1,2-Dibromo-3-chloropropane | 3 |  | 217,563 | 159,744 | **26.6** |  | 15,390 | 30,664 | **99.2** |
| 18 | Chloropropene | 1,1-Dichloropropene | 3 |  | 150,469 | 168,107 | **11.7** |  | 63,435 | 42,232 | **33.4** |
| 19 | (Exp-DI: II + III | cis-1,3-Dichloropropene | 3 |  | 173,021 | 168,107 | **2.84** |  | 47,909 | 42,232 | **11.8** |
| 20 | Exp-SPME: I + II + III) | trans-1,3-Dichloropropene | 3 |  | 180,830 | 168,107 | **7.04** |  | 41,349 | 42,232 | **2.14** |
| 21 |  | 1,1-Dichloroethylene | 2 |  | 98,643 | 116,176 | **17.8** |  | 22,595 | 58,476 | **159** |
| 22 | Chloroethene | trans-1,2-Dichloroethylene | 2 |  | 137,005 | 116,176 | **15.2** |  | 44,783 | 58,476 | **30.6** |
| 23 | (Exp-DI: II + III | cis-1,2-Dichloroethylene | 2 |  | 104,683 | 116,176 | **11.0** |  | 34,524 | 58,476 | **69.4** |
| 24 | Exp-SPME: All compounds) | Trichloroethylene | 2 |  | 180,219 | 116,176 | **35.5** |  | 75,036 | 58,476 | **22.1** |
| 25 |  | Tetrachloroethylene | 2 |  | 60,328 | 116,176 | **92.6** |  | 89,111 | 58,476 | **34.4** |
| 26 |  | Benzene | 6 |  | 372,099 | 346,953 | **6.76** |  | 126,383 | 136,828 | **8.26** |
| 27 |  | Chlorobenzene | 6 |  | 114,275 | 346,953 | **204** |  | 124,752 | 136,828 | **9.68** |
| 28 |  | Bromobenzene | 6 |  | 296,751 | 346,953 | **16.9** |  | 108,912 | 136,828 | **25.6** |
| 29 |  | 1,3-Dichlorobenzene | 6 |  | 431,589 | 346,953 | **19.6** |  | 142,393 | 136,828 | **3.91** |
| 30 |  | 1,4-Dichlorobenzene | 6 |  | 441,353 | 346,953 | **21.4** |  | 150,407 | 136,828 | **9.03** |
| 31 |  | 1,2-Dichlorobenzene | 6 |  | 433,475 | 346,953 | **20.0** |  | 135,053 | 136,828 | **1.31** |
| 32 |  | 1,2,4-Trichlorobenzene | 6 |  | 457,152 | 346,953 | **24.1** |  | 138,511 | 136,828 | **1.21** |
| 33 |  | 1,2,3-Trichlorobenzene | 6 |  | 467,779 | 346,953 | **25.8** |  | 132,429 | 136,828 | **3.32** |
| 34 | Aromatic | Toluene | 7 |  | 384,687 | 399,669 | **3.89** |  | 173,839 | 158,151 | **9.02** |
| 35 | (Exp-DI: All compounds | 2-Chlorotoluene | 7 |  | 272,410 | 399,669 | **46.7** |  | 167,819 | 158,151 | **5.76** |
| 36 | Exp-SPME: II + III + IV) | 4-Chlorotoluene | 7 |  | 363,124 | 399,669 | **10.1** |  | 171,443 | 158,151 | **7.75** |
| 37 |  | Ethylbenzene | 8 |  | 327,795 | 452,384 | **38.0** |  | 230,317 | 179,474 | **22.1** |
| 38 |  | m-Xylene | 8 |  | 840,023 | 452,384 | **46.1** |  | 364,545 | 179,474 | **50.8** |
| 39 |  | o-Xylene | 8 |  | 364,964 | 452,384 | **24.0** |  | 169,456 | 179,474 | **5.91** |
| 40 |  | Styrene | 8 |  | 310,801 | 452,384 | **45.6** |  | 168,866 | 179,474 | **6.28** |
| 41 |  | Isopropylbenzene | 9 |  | 514,803 | 505,100 | **1.88** |  | 191,197 | 200,796 | **5.02** |
| 42 |  | n-Propylbenzene | 9 |  | 473,256 | 505,100 | **6.73** |  | 191,571 | 200,796 | **4.82** |
| 43 |  | 1,3,5-Trimethylbenzene | 9 |  | 440,474 | 505,100 | **14.7** |  | 207,969 | 200,796 | **3.45** |
| 44 |  | 1,2,4-Trimethylbenzene | 9 |  | 426,524 | 505,100 | **18.4** |  | 203,363 | 200,796 | **1.26** |
| 45 |  | tert-Butylbenzene | 10 |  | 414,009 | 557,815 | **34.7** |  | 213,061 | 222,119 | **4.25** |
| 46 |  | sec-Butylbenzene | 10 |  | 559,816 | 557,815 | **0.36** |  | 190,895 | 222,119 | **16.4** |
| 47 |  | p-Isopropyltoluene | 10 |  | 615,956 | 557,815 | **9.44** |  | 207,693 | 222,119 | **6.95** |
| 48 |  | n-Butylbenzene | 10 |  | 605,130 | 557,815 | **7.82** |  | 197,134 | 222,119 | **12.7** |
| 49 |  | Naphtalene | 10 |  | 751,025 | 557,815 | **25.7** |  | 150,269 | 222,119 | **47.8** |
|  | Mean |  |  |  |  |  | **27.5** |  |  |  | **30.3** |
|  | SD |  |  |  |  |  | **34.2** |  |  |  | **45.3** |
|  |  |  |  |  |  |  |  |  |  |  |  |
| ^a^ Predictive equations ((1) Slopes and (2) intercepts) developed for 13 arbitrary groups (codes) in Table 6S are used: | | | | | | | | | | |  |
| **Exp-DI:** (1) Slope: Eqn (I + III) = 27,054, Egn (II + III) = 51,931, Egn (All compounds) = 52,715 | | | | | | | | | | | |
|  | (2) Intercept: Eqn (I + III) = 78,582, Egn (II + III) = 12,313, Egn (All compounds) = 30,661 | | | | | | | | | |  |
| **Exp-SPME:** (1) Slope: Eqn (I) = 6,309, Egn (I + II + III) = 9,818, Egn (All compounds) = 23,642, Egn (II + III + IV) = 21,323 | | | | | | | | | | |  |
|  | (2) Intercept: Eqn (I) = 11,738, Egn (I + II + III) = 12,777, Egn (All compounds) = 11,192, Egn (II + III + IV) = 8,892 | | | | | | | | | |  |
| ^b^ The best projected RFs are derived by taking the min PD value for each compound (out of 49) after testing against 13 equations linear regression equations (between the number of carbon (X axis) and actual RF values (Y axis)). | | | | | | | | | | | |
| ^c^Percent difference (PD) = **\|**(RF (Projected) - RF (Actual)**\|** / RF(Actual) * 100.  ^d^Best fit equation for a given chemical group. | | | | | | | |  |  |  |  |
|  |  |  |  |  |  |  |  |  |  |  |  |

[A] Carbon number

[B] Molecular weight

[C] Boiling point

[C] Boiling point

Fig. 1S. The correlations between RF values and key variables (A. carbon number, B. molecular weight, and C. boiling point) in Ahn et al. [4].
